# Tolerance of anaerobic conditions caused by flooding during germination and early growth in rice (*Oryza sativa* L.)

**DOI:** 10.3389/fpls.2013.00269

**Published:** 2013-07-23

**Authors:** Berta Miro, Abdelbagi M. Ismail

**Affiliations:** Crop and Environmental Sciences Division, International Rice Research InstituteManila, Philippines

**Keywords:** anaerobic germination, alcoholic fermentation, ALDH, pyruvate dehydrogenase bypass, hypoxia, direct seeding, flooding, submergence tolerance

## Abstract

Rice is semi-aquatic, adapted to a wide range of hydrologies, from aerobic soils in uplands to anaerobic and flooded fields in waterlogged lowlands, to even deeply submerged soils in flood-prone areas. Considerable diversity is present in native rice landraces selected by farmers over centuries. Our understanding of the adaptive features of these landraces to native ecosystems has improved considerably over the recent past. In some cases, major genes associated with tolerance have been cloned, such as *SUB1A* that confers tolerance of complete submergence and *SNORKEL* genes that control plant elongation to escape deepwater. Modern rice varieties are sensitive to flooding during germination and early growth, a problem commonly encountered in rainfed areas, but few landraces capable of germination under these conditions have recently been identified, enabling research into tolerance mechanisms. Major QTLs were also identified, and are being targeted for molecular breeding and for cloning. Nevertheless, limited progress has been made in identifying regulatory processes for traits that are unique to tolerant genotypes, including faster germination and coleoptile elongation, formation of roots and leaves under hypoxia, ability to catabolize starch into simple sugars for subsequent use in glycolysis and fermentative pathways to generate energy. Here we discuss the state of knowledge on the role of the PDC-ALDH-ACS bypass and the ALDH enzyme as the likely candidates effective in tolerant rice genotypes. Potential involvement of factors such as cytoplasmic pH regulation, phytohormones, reactive oxygen species scavenging and other metabolites is also discussed. Further characterization of contrasting genotypes would help in elucidating the genetic and biochemical regulatory and signaling mechanisms associated with tolerance. This could facilitate breeding rice varieties suitable for direct seeding systems and guide efforts for improving waterlogging tolerance in other crops.

## Introduction

Waterlogging and floods cause considerable yield losses of major food crops worldwide. For dryland cereals such as maize, wheat, rye and barley, yield losses in rainfed and irrigated areas can reach 20% in some regions (Setter and Waters, 2003). Among cereal crops, rice is unique in being capable of growing well in waterlogged and submerged soils because of its well-developed aerenchyma system that facilitates aeration of the roots and the rhizosphere, thus alleviating most of the stresses experienced under low oxygen (Setter et al., 1997; Jackson and Ram, 2003). However, this escape mechanism is inadequate when floods are partially covering the plants for longer durations of a few weeks to months, as in stagnant, semi-deep (25–50 cm) and deepwater areas (over 50 cm to several meters), or when floods are transient but cause complete inundation for shorter durations of up to 2 weeks as in flash-flood-affected areas. These types of floods are common in rainfed lowlands and flood-prone areas worldwide, and cause considerable losses in grain production each year (Singh et al., 2009, 2011; Mackill et al., 2012; Ismail et al., 2013; Colmer et al., 2014). Floods are therefore considered major challenges for rice production, especially in South and Southeast Asia, where the majority of the world's rice farmers live and depend on rice and rice-based farming as their major source of food, income and livelihood. Moreover, the impacts of these floods seem to be worsening in recent years, due to effects commonly attributed to climate change, such as sea-level rise, the uneven distribution of rains and periodic changes in frequencies and intensities of floods caused by extreme weather events (Coumou and Rahmstorf, 2012). Nonetheless, the enormous diversity and plasticity in adaptation to contrasting hydrological conditions, ranging from aerated soils in uplands to areas with water depths exceeding 5 m in deepwater areas, has made rice one of the most amenable crops for genetic manipulation to develop varieties suitable for excess water conditions (Ismail and Mackill, 2013; Kirk et al., 2014). Several landraces that can withstand different types of floods have been identified and characterized (Table 1). Some of these landraces have subsequently been used to breed high yielding modern varieties (Mackill et al., 2012; Ismail et al., 2013).

**Table 1:** **Examples of rice genotypes identified as tolerant or sensitive of different types of floods and main traits associated with tolerance**.

**Developmental stage**	**Type of flood**	Tolerant varieties^*^	Sensitive varieties^*^	**Main traits associated with tolerance**	**Major QTLs/Genes**	**References**
Germination and early seedling growth	Flash floods/submergence Waterlogging	Khao Hlan On	IR42	− Fast germination and coleoptile elongation	*qAG7.1*	Ismail et al., 2009, 2012; Angaji et al., 2010; Septiningsih et al., 2013
		Ma-Zhan (Red)	IR64	− Ability to break down and use starch under low O_2_	*qAG-9-2*	
		Khaiyan	FR13A			
		Kalonchi		− High anaerobic respiration to avoid energy crises when oxygen is low		
		Kharsu				
		Nanhi				
Vegetative stage	Flash flood	FR13A	IR42	− Reduced elongation	*SUB1A-1*	Jackson and Ram, 2003; Sarkar et al., 2006; Xu et al., 2006; Mackill et al., 2012; Winkel et al., 2012; Ismail et al., 2013
	Complete submergence	FR43B	IR64	− Slow carbohydrate consumption during submergence		
		Kurkaruppan				
		Goda Heenati		− Underwater photosynthesis		
		Thavalu		− Chlorophyll retention underwater		
				− Fast recovery	
	Stagnant, medium-deep (30–50 cm), longer duration (weeks to months)	IRRI 119	Swarna	− Partial, slow elongation of the shoot	Not genetically characterized	Singh et al., 2011
		IRRI 154	IR64, most modern varieties	− High tillering ability underwater		
				− Strong culms, resistant to lodging		
				− Sufficient leaf area above water		
				− Large fertile panicles		
	Deep-water (>50 cm to > 5 m)	Jalmagna	All modern lowland varieties	− Fast internode elongation with rising water (> 20 cm per day)	*SNORKEL1*	Catling, 1992; Hattori et al., 2007, 2009
		Baisbish			*SNORKEL2*	
		Rayada 16-3		− Sufficient leaf area above water		
		Nang Dum To		− Kneeing ability when water recedes		
		Sudu Gries		− Large fertile panicles		

* *Tolerant and sensitive varieties in the table are representative examples. Modified from Ismail and Mackill (2013)*.

Notwithstanding its tolerance of waterlogging and shallow floods during the vegetative stage, rice is extremely sensitive to anaerobic conditions during germination (anaerobic germination) and early growth of the embryo (Yamauchi et al., 1993; Ismail et al., 2009; Angaji et al., 2010). Rice seeds can germinate, and to some degree, extend their coleoptiles under hypoxic and even anoxic conditions, but fail to develop roots and leaves (Taylor, 1942; Biswas and Yamauchi, 1997; Ella and Setter, 1999). Tolerance of anaerobic conditions at these early stages is a prerequisite for effective direct-seeded rice in rainfed and flood-affected areas. Soil waterlogging or flooding can be encountered when it rains immediately after seeding or when the land is not well-levelled in irrigated areas (Kirk et al., 2014). In either case severe reductions in or failure of crop establishment can be experienced (Ismail et al., 2012). Varieties that can germinate in flooded soils could be beneficial for direct-seeded systems in these areas and even for intensive irrigated systems, where early flooding can suppress weeds (Ismail et al., 2012). This will consequently result in enormous savings in production costs as opposed to when rice is transplanted. It can also reduce the cost of manual or mechanical weeding or the use of hazardous chemicals for weed control.

Considerable progress has been made in understanding the genetics and physiology of tolerance of flooding during vegetative stage in rice and also during germination as compared with other crops, and this has been comprehensively reviewed over the recent past (e.g., Jackson and Ram, 2003; Magneschi and Perata, 2009; Bailey-Serres and Voesenek, 2010; Bailey-Serres et al., 2010, 2012; Ismail et al., 2012, 2013; Colmer et al., 2014). Genetic variation in the ability to germinate and establish in flooded soils has recently been observed in rice, and a few landraces were identified that are tolerant (Angaji et al., 2010). Here, we attempt to review some of the main physiological and molecular mechanisms studied over the past few decades that can likely explain this genetic variability in tolerance of flooding during germination within rice. We focus on alternative pathways and genes that are suggested to play a role in situations when energy crises arise in germinating seeds or other systems under low oxygen stress. Examples and evidences from studies conducted on rice and other plant species are highlighted in an attempt to recognize the traits and pathways possibly involved in tolerance. We also attempt to pinpoint gaps in knowledge to direct future studies on germination under water. Adequate understanding of the adaptive traits and pathways will help in designing effective breeding strategies to develop tolerant rice varieties for direct-seeding systems, and could guide efforts to improve tolerance of waterlogging in other crop species facing similar challenges.

## Plant responses to flooding during germination

Several adverse conditions occur in the root zone when plants germinate under water: oxygen becomes scarce hindering root respiration and growth and gases such as CO_2_ and ethylene build up. Low oxygen causes a reduction in root growth and function, thus reducing nutrient and water uptake. In addition, several phytotoxic substances such as reduced iron (Fe^+2^), manganese (Mn^+2^), hydrogen sulphide (H_2_S), and intermediates of anaerobic carbon metabolism—e.g., organic acids—accumulate to toxic concentrations. These alter the soil pH and further affect the availability and uptake of nutrients by the plant. Together, these changes cause injury to roots and the whole plant, and can lead to plant death in severe cases (Drew and Lynch, 1980; Kirk et al., 2014). Even though some genetic diversity in waterlogging tolerance has been found within some crops, as in maize, wheat, barley and rye, this diversity has not been sufficiently exploited through breeding (Drew, 1997; Setter and Waters, 2003).

Plants adapted to waterlogged or submerged conditions during germination and establishment develop different strategies to cope with these adversities. These tactics include morphological adjustments such as mesocotyl root development in *Echinochloa* (Everard et al., 1991), adventitious root development in *Rumex palustris* (Visser et al., 1997), petiole elongation also in *Rumex palustris* (Voesenek et al., 1990) and *Ranunculus sceleratus* (Horton, 1993), and coleoptile elongation in some rice varieties (Pearce and Jackson, 1991; Ismail et al., 2009). The discovery of rice genotypes with better tolerance of flooding during germination facilitated mechanistic studies to decipher some of the traits likely associated with tolerance (Ismail et al., 2009; Angaji et al., 2010; Ella et al., 2010, 2011; Septiningsih et al., 2013). Furthermore, good progress was made in developing breeding lines that are more suitable for direct-seeded systems. Major QTLs (quantitative trait loci) associated with tolerance of flooding during germination have been recently identified and are being targeted for cloning and for use in marker-assisted breeding (Angaji et al., 2010; Septiningsih et al., 2013). Despite this progress, little is known about the molecular basis of tolerance of low oxygen stress in these contrasting rice genotypes.

**Figure 1:**
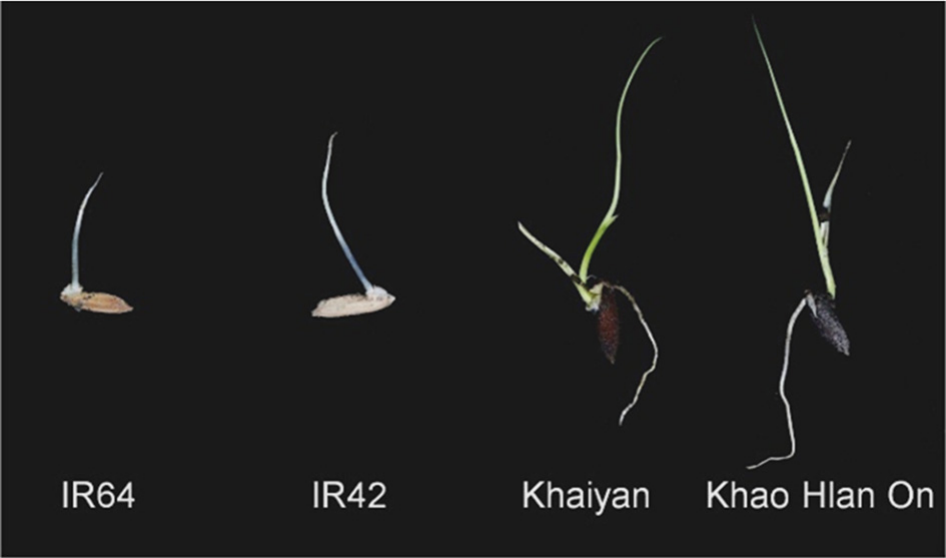
**Left to right: seedlings of sensitive varieties “IR64” and “IR42” and tolerant landraces “Khao Hlan On” and “Khaiyan” 7 days after sowing in soil and flooding with 10 cm of water (from image collection of the International Rice Research Institute (IRRI) publicly available at Flickr http://www.flickr.com/photos/ricephotos/2199544108/in/photostream/)**.

When flooding occurs just after direct seeding, tolerant rice genotypes germinate faster and their coleoptiles grow at a relatively faster rate to emerge from flooded soils. These genotypes are also capable of forming roots and leaves in shallow water depths (Ismail et al., 2009; Angaji et al., 2010; Figure 1). Some progress has been made in unraveling the metabolic processes that are likely associated with tolerance. These include the ability to initiate and maintain carbohydrate catabolism in germinating seeds, anaerobic respiration to sustain energy supply and maintenance of cellular extensibility of the growing embryo. As the seedling elongates to more aerated zones, aerenchyma tissue develops to provide oxygen for the submerged plant parts, especially to roots, through what is frequently referred to as the snorkel effect (Alpi and Beevers, 1983; Kawai and Uchimiya, 2000). Progressive detoxification of oxygen radicals generated in seeds and of other toxins that develop in anoxic soils also helps in preventing further injury (Ismail et al., 2009, 2012; Colmer et al., 2014; Kirk et al., 2014).

### Coleoptile elongation; an escape strategy

Rice can germinate under hypoxic or anoxic conditions, but only tolerant genotypes have the ability of fast coleoptile elongation and root formation under submerged conditions in the field (Ismail et al., 2009) (Figure 1). Conversely, the coleoptile growth is slow in sensitive genotypes, and they fail to develop further. Coleoptile elongation is usually targeted for selection of tolerant rice genotypes germinating under submergence because it is easy to phenotype. Coleoptile length increases mainly through cell elongation. Cell division is active during the first 48 h of submergence, and that is the period when oxygen is mostly required (Atwell et al., 1982). Since cellular expansion consumes less energy than cell division, the latter is the main process governing elongation under anoxia (Atwell et al., 1982; Magneschi and Perata, 2009). However, no apparent variation in growth has been found between seedlings of tolerant and sensitive genotypes when seeds were pre-soaked or primed through soaking in water and dehydration before sowing and submergence (Ella et al., 2011). Presumably most rice genotypes are capable of initiating germination but cannot elongate further, probably because of bottlenecks in using energy reserves when oxygen is limiting.

Coleoptile elongation is thought to be related to the role of specific expansins under anaerobic conditions for their action in cell wall loosening (Huang et al., 2000; Choi et al., 2003; Ismail et al., 2009; Magneschi and Perata, 2009). Firstly, *EXPA2* and *EXPA4* were found to be expressed in submerged coleoptiles but not in aerobic or anoxic ones (Huang et al., 2000). A later study correlated *EXPA4* with coleoptile and mesocotyl length (Choi et al., 2003). Moreover, *EXPA7* and *EXPB12* were observed to be highly expressed in elongating coleoptiles under anoxia (Lasanthi-Kudahettige et al., 2007). This would agree with previous results that *EXPA2* and *4* are specific to hypoxia and therefore were not detected in the anoxic study by Lasanthi-Kudahettige et al. (2007). Another 3 expansins were also reported to be induced under submergence by Takahashi et al. (2011). These expansins *EXPA1*, *EXPB11*, and *EXPB17* were differentially expressed in the *rad* mutant in comparison with the control, and were affected by deficiency in ADH1. Therefore, these expansins may also be related to elongation under submergence. The importance of expansins for coleoptile extension under submergence seems clearly justified in all these studies. However, all expansins seem equally important and their activity may depend on the kind of stress or genotype being studied.

Another group of enzymes known to be associated with cell wall extensibility are peroxidases (EC 1.11.1), whose activities reduce cell extensibility (Ismail et al., 2009 and citations therein). The association of increased activities of peroxidases with reduction in elongation has been reported in some species, as in mung bean (Goldberg et al., 1987), peanuts (Zheng and van Huystee, 1992) and rice (Ismail et al., 2009). In the latter, higher peroxidase activity was reported to be negatively correlated with seed germination and seedling survival under submergence, and peroxidase concentration was significantly higher in the sensitive genotypes FR13A and IR42. Peroxidase activities, especially the cell bound forms, are negatively correlated with coleoptile elongation (Ismail et al., 2009). Yet other classes of structural proteins and enzymes were recently implicated. The action of tubulin a-1 chain (TUBA1) and actin depolymerizing factor 4 (ADF4) were suggested to be involved in fast coleoptile elongation under anaerobic conditions, as both genes are upregulated under anoxia during germination in rice (Sadiq et al., 2011). Tubulins are involved in the formation of microtubules and are important for both cell division and elongation, and ADF4 is involved in regulating actin assembly (Mayer and Jürgens, 2002; Augustine et al., 2008; Sadiq et al., 2011); therefore both are speculated to play a role in fast growth of the coleoptiles under low oxygen. However, further studies are required to ascertain the actual roles of these genes in coleoptile growth and tolerance of hypoxia or anoxia.

#### Phytohormones involved in germination and coleoptile elongation under submergence

Mechanisms of tolerance of complete submergence during vegetative stage are fairly established following the discovery of the *SUB1* genes and their further characterization (Bailey-Serres and Voesenek, 2010; Bailey-Serres et al., 2010). *SUB1A*, an ethylene response factor (ERF; Xu et al., 2006) induces “quiescence” state marked by reduction of elongation and slowing of relevant metabolic processes (Bailey-Serres et al., 2010; Schmitz et al., 2013). In addition to *SUB1A*, some wild rice genotypes belonging to the C-genome group showed a similar tolerance mechanism involving a *SUB1* ortholog with high similarity to *SUB1C* (Niroula et al., 2012). Recently, another two ERFs, *SNORKEL1* and *SNORKEL2*, that control fast elongation under deepwater conditions were cloned and characterized (Hattori et al., 2009). Differential response to the content of the gaseous hormone ethylene thus seem to be an important aspect of submergence tolerance, either through inhibiting elongation or through promoting active growth. Indeed ethylene was shown to be involved in internode elongation in deepwater rice (Metraux and Kende, 1983). Coleoptile elongation was also reported to be regulated by ethylene (Atwell et al., 1982; van der Straeten et al., 2001; Zhou et al., 2002; Jackson, 2008). However, an ethylene-independent anoxia response was proposed (Pearce and Jackson, 1991; Pearce et al., 1992), since ethylene and its precursor 1-aminocyclopropane-1-carboxylic-acid (ACC) would not function without oxygen (reviewed in Bailey-Serres et al., 2010). While coleoptile elongation under submergence does not occur under anoxic, but rather under hypoxic conditions in the field, the action of ethylene may still be relevant in such conditions. Other studies suggested that the importance of ethylene may be genotype dependent (Dubois et al., 2011). Ismail et al. (2009) argued that the involvement of ethylene in coleoptile elongation under submergence probably occurs at the later stages, after active cell division and when cell expansion is predominant. Ethylene could therefore become mainly involved in coleoptile elongation when they are in contact with relatively more aerated layers of the floodwater (Ismail et al., 2012).

The phytohormones ABA and GA also seem to play important roles in submergence tolerance during germination. A recent *in silico* analysis identified patterns of co-expression of different genes under anoxia (Mohanty et al., 2012). This revealed ABA as a positive regulator of rice germination under submergence and its plausible interaction with ethylene for coleoptile elongation. The authors also speculated that GA may be related to inhibition of the coleoptile response to submergence during germination. These results are an apparent contradiction with the known functions of ABA and GA, which are involved in seed dormancy (Hoffmann-Benning and Kende, 1992) and seed germination, respectively. A QTL for tolerance of anaerobic conditions during germination (*qAG12*, Septiningsih et al., 2013) contains candidate genes that are possibly involved in promoting ABA synthesis during early stages of seed development. However, these genes–containing PIL5 (LOC_Os12g40700) and bHLH (LOC_Os12g40730) motifs, were identified within the QTL *qSD12* for seed dormancy (Gu et al., 2010). Both QTLs are located close to another QTL for GA (*qGAR-12*) and for seedling height (*qSPH-12*; Septiningsih et al., 2013). The authors suggested that the QTL for GA may regulate the QTL for anaerobic germination, since GA could promote coleoptile elongation under submergence according to Horton (1991). They also suggested that alleles governing the QTL for anaerobic germination may be different from those of *qSD12* since an opposite function for ABA is expected. Further research on the *qAG12* will identify the genes governing this QTL and inform on the complex role of plant hormones in anaerobic germination. Whether the hormones exchange roles as suggested by Mohanty et al. (2012) or that different alleles with opposite function govern these QTLs as suggested by Septiningsih et al. (2013) will need to be unraveled. Based on Mohanty et al. (2012), if anaerobic germination is positively regulated by ABA, the genotype Nipponbare they studied should not follow an escape strategy, but rather become quiescent under anoxic conditions. Additional results of Mohanty et al. (2012) suggest that sugar, oxygen and ethylene are part of the same activation cascade and also involve ABA and expansins during germination under flooded conditions. A similar network, which also included CIPK15 was invoked by Bailey-Serres and Voesenek (2010), except for the activation of coleoptile elongation. If the ethylene dependent and independent pathways could be linked at the ABA level and not at the shoot elongation level, then the two networks would be rather similar. However, ABA is linked to seed dormancy, suggesting high levels of the hormone would send the seed in a quiescence mode rather than activating growth of the embryo.

These divergent evidences observed in different studies could probably be partially related to experimental conditions, such as the seeds being tested under different oxygen levels or the use of varieties with variable tolerance. For example, varieties with different coping strategies may have different signaling responses involving antagonistic hormones. Such environmental and/or genetic variability could lead to diverse sets of data that should be integrated to predict the most relevant mechanisms. New studies should bring more insights into the regulatory and signaling pathways involving these hormones in different genetic backgrounds adapted to specific environments, yet contrasting in germination and early growth under submergence. According to Ismail et al. (2009, 2012) the roles of different hormones under submergence are difficult to interpret without a standardized phenotyping strategy that addresses the genetic and environmental variables such as O_2_ and CO_2_ concentrations, temperature and floodwater conditions. Light regime is also an important aspect of the content and interplay between ABA and GA both of which, despite classical associations with dormancy and germination, respectively, are still not definitively associated with the respective process in all plants.

Oxygen deprivation under submergence is one of the key elements that trigger different coping responses to overcome the stress. Direct oxygen signaling, sugar, calcium and pH status are some of the rudiments thought to play major roles in the signaling cascade during germination under submergence (reviewed in Bailey-Serres et al., 2012). Signaling molecules such as nitric oxide (NO), peroxide and reactive oxygen species are also linked to these processes (Finkelstein, 2010; Liu et al., 2010; Hill, 2012). Clearly, several metabolic processes are involved in the tolerant phenotype in rice, which are coordinated in a manner that facilitates germination and fast growth of the embryo to emerge from flooded soils. Since both ethylene-dependent and -independent mechanisms activate a signaling cascade, whether these are truly two independent mechanisms or they are simultaneously coordinated at a specific level remains to be known. Further work is necessary to untangle the regulatory and signaling mechanisms that facilitate these two processes.

## Other traits associated with anaerobic germination

### Selectable traits

One of the main areas of research in anaerobic germination of rice is the identification of traits indicative of the tolerance phenotype. The main trait used is seedling survival after 21 days of submergence under 10 cm water head. Surviving seedlings are those that emerge from the water by fast germination and coleoptile elongation (Ismail et al., 2009; Angaji et al., 2010). Another important trait is the activity of enzymes such as the α-amylases, anaerobic respiration enzymes and others from the TCA cycle, in the anaerobic pathways. Colorimetric reactions for many enzymes including dehydrogenases and peroxidases have been developed for high-throughput screening, making enzyme analysis a valid screening methodology for anaerobic germination. Moreover, new digital imaging techniques interlinking colorimetric assays with imaging spectroscopy make the screening much more straightforward.

Seed longevity is also related to anaerobic germination as shown by Ella et al. (2010) and Septiningsih et al. (2013). The first paper reported a decrease in germination under submergence when using older seeds. It also suggested increased lipid peroxidation and decreased superoxide dismutase and catalase activities as the reasons for the reduction in seed viability. Septiningsih et al. (2013) discussed the co-location of QTLs for anaerobic germination with QTLs related to seed ageing traits and suggested a relation between seed dormancy, longevity and germination under submergence. Traits associated with seed aging such as the extent of lipid peroxidation, could potentially be used as markers for indirect selection. Other morphological traits worth the attention are coleoptile diameter, days to emergence of first leaf, and the first leaf width and length. Also root characteristics such as root length, diameter, secondary root development and root hairs may be monitored. These traits may be relevant when studying genotypes with intermediate tolerance or when comparing different groups such as *indica*, *aus*, and temperate and tropical *japonica*. However, their relevance as selectable traits still needs to be assessed especially in relation to survival and/or coleoptile elongation.

### Enzymes related to energy production under low oxygen

Limitations of energy supply under oxygen deficient conditions caused by submergence are a major bottleneck for seed germination and seedling survival. The main adjustment is the shift from the aerobic to the anaerobic metabolism. The anaerobic fermentative processes normally generate 2–3 ATP per molecule of glucose, compared with the 36–38 molecules generated through aerobic metabolism (Fox et al., 1994; Greenway and Setter, 1996). It is also known that Pasteur Effect, involving accelerated rates of carbohydrate catabolism can provide up to 37.5% of the ATP generated under aerobic conditions in the coleoptiles of tolerant rice genotypes (Gibbs and Greenway, 2003). Hence, supplementing the energy supply through aerobic metabolism via respiration with that from anaerobic metabolism may be useful under hypoxic conditions for coleoptile growth. Some enzymes active under anoxia can use PPi instead of ATP to adapt to the low-energy environment (Carystinos et al., 1995). This suggestion was supported by the results of Huang et al. (2005a), showing up-regulation of nucleoside diphosphate kinase under anoxia, an enzyme associated with coleoptile elongation under submergence.

Another study implicating enzymes of the anaerobic pathway in rice coleoptile growth under anoxic conditions assessed the genetic variation in ATP production in six different rice genotypes; two tolerant (“Khao Hlan On” and “Khaiyan”; Ismail et al., 2009), two moderately tolerant (“Nipponbare” and “Kinmaze”) and two mutant lines (*rad* lacking the ADH gene and a *PDC* insertion mutant). The study found that under both hypoxic and anoxic conditions an ATP production rate of about 10% that of the aerated conditions was maintained in the tolerant and moderately tolerant lines. The *PDC* insertion mutant maintained close to 7% and the *rad* mutant only 3.7% of the ATP produced under normoxia (Edwards et al., 2012).

### Enzymes related to cytoplasmic acidosis

At the cellular level, changes in cytoplasmic pH have been considered important for cell integrity and function, energy consumption and the activation or inactivation of enzymes involved in anaerobic metabolism (Magneschi and Perata, 2009). These changes have also been considered an adaptive advantage of varieties tolerant of anoxia vs. sensitive varieties (reviewed in Greenway and Gibbs, 2003). Studies comparing pH changes under anoxia in rice and wheat reported that the cytoplasmic pH quickly recovered from low pH, and is therefore somehow regulated (Menegus et al., 1991; Kulichikhin et al., 2009). Vacuolar pH increased in tolerant rice (Menegus et al., 1991; Kulichikhin et al., 2007, 2009), but not in sensitive wheat roots under submergence (Menegus et al., 1991; Kulichikhin et al., 2007). The usual pH under aerobic conditions is about 5.6 in vacuoles of rice coleoptiles, but it becomes increasingly basic under anoxic conditions, reaching 6.0 after 14 h (Menegus et al., 1991). This pH shift in the vacuole in particular was suggested to favor a reduction in cellular energy consumption by decreasing the pH difference between the cytoplasm and the vacuole (Menegus et al., 1993). This is in agreement with later results suggesting the higher vacuolar pH is a response to a new metabolic state of energy crisis, and it is not related to cell injury under anoxia (Felle, 2005; Kulichikhin et al., 2009). Kulichikhin et al. (2009) observed that tolerant tissues under anoxia maintain a stable pH for up to 4 days. Thus, the ability to maintain the cytoplasmic pH and avoid acidosis may be an important component of tolerance of low oxygen stress. Davies et al. (1993) reported that cytoplasmic acidification as a consequence of anoxia would increase the release of energy through PPi, but would have an opposite effect on ATP synthesis. Both energy and pH maintenance in submerged rice coleoptiles have been shown to be directly related to ethanolic fermentation rates (Gibbs et al., 2000), however, association with genetic variation in germination under anaerobic conditions within rice awaits further studies.

A reduction in cytoplasmic pH has been associated with the lactic acid fermentation pathway, where lactate is produced from pyruvate through the activity of lactate dehydrogenase (LDH, EC 1.1.1.27.). The role of lactate in anoxic rice coleoptiles has been discussed and some studies suggested that it is related to a pH decrease that would then activate alcoholic fermentation as in maize root tips (Roberts et al., 1992). This reaction would generate protons that decrease the cytosolic pH by less than 1 unit. However, this role was later rebutted since lactate concentrations alone could not account for the total pH decrease in either rice coleoptiles (Menegus et al., 1991) or maize root tips (Saint-Ges et al., 1991). The ratio of succinate:lactate was also found to be relevant to the final cytoplasmic acidosis and could differentiate between tolerant and non-tolerant species depending on their ability to produce more succinate than lactate to survive anoxia (Menegus et al., 1989). Further, proton-consuming reactions in rice, such as glutamate decarboxylation could maintain the cytoplasmic pH (Menegus et al., 1989). Glutamate decarboxylation was shown to occur under anoxia in rice (Fan et al., 2003). Felle (2005) and Wang et al. (2007) discussed the role of nitrate as a proton acceptor and thus as a pH regulator. Reggiani et al. (1993, 1995) suggested that nitrate is assimilated in rice seeds germinating under anaerobic conditions. This hypothesis was supported by the studies of Fan et al. (1997) in rice coleoptiles and recently, Greenway et al. (2012) reported that nitrate is converted to ammonium in anoxic rice coleoptiles, which would maintain the pHstat. However, nitrate is quickly reduced in anaerobic soils and its implication in the internal pH balance of rice under anoxic stress is not clear (Greenway et al., 2012). The authors also attempted to assess if a lower pH would compromise plant survival or if anaerobic metabolism would be a durable supplement as suggested by Huang et al. (2005b). However, they found that the pH decrease was balanced by nitrate reduction to ammonium, organic acid pHstat and decreased permeability coefficient of the plasma membrane for protons. The implications of pH regulation under flooding have been reported in various studies, showing that tolerant species are capable of controlling cytoplasmic and vacuolar pH, and can therefore survive longer periods of anoxia (Felle, 2005; Kulichikhin et al., 2009; Greenway et al., 2012). However, the role of this pH regulation in causing higher tolerance within rice needs to be established in contrasting genotypes. Since different substrates may be involved in cytoplasmic acidosis and pH status regulation in submerged rice coleoptiles, a holistic analysis of all reactions that affect cellular pH under anoxia is necessary.

### Haemoglobins in the HB/NO cycle

The nitrite oxidation product is nitric oxide (NO), which is linked to haemoglobins (Hb) through the Hb/NO cycle. Hypoxic stress activates Hb, probably through nitrite and oxygen levels that increase NO, which may then reduce metabolic activity in stressed cells (Hill, 2012). The NO and Hb are interlinked under hypoxia or anoxia in rice plants and may provide an alternative pathway for the electron transport chain under limiting oxygen conditions (Igamberdiev and Hill, 2004). Furthermore, the Hb/NO cycle maintains energy levels under anoxia and Hb may activate some signaling cascade through NO and ethylene, which in turn could trigger the development of root aerenchyma (Igamberdiev and Hill, 2004). NO may also act as an electron acceptor under oxygen deprivation, since Hb/NO cycle and glycolysis showed similar ATP production rates (15–17 nmol min^−1^ mg^−1^ protein) and that these were 3–5% of the aerobic ATP generated in the mitochondria (Stoimenova et al., 2007). The authors also proposed that ATP is synthesized from succinate. Both Igamberdiev and Hill (2004) and Stoimenova et al. (2007) compared rice with other species such as maize and barley, but there have been no extensive studies within rice on Hb and NO, particularly looking at contrasting rice genotypes under anoxia or hypoxia.

## Adjustments of carbohydrate metabolism under low oxygen

Carbohydrate metabolism is strongly inhibited when oxygen is limiting, especially the steps involved in the breakdown of starch into simple sugars for use in glycolysis, mainly because most of the enzymes involved are less active under low oxygen. A major reason for this inhibition is the activation of energy conserving steps by changing enzyme activities. However, some of these enzymes are active in rice genotypes that are tolerant of hypoxia during germination; among them are α-amylases (especially *RAmy3D*), sucrose synthase and aldolase, but inhibited in sensitive genotypes (Ismail et al., 2009). The second limiting step causing energy crises under low oxygen stress is the breakdown of pyruvate to generate energy through the TCA cycle. This cycle is inhibited by lack of oxygen, which acts as a terminal electron acceptor. Under such conditions, most plants, including rice, activate alternative pathways to supply substrates and sustain energy generation through glycolysis. Fermentative metabolism or anaerobic respiration in submerged coleoptiles uses alcohol, lactate and alanine fermentation pathways to regenerate NAD^+^ required for glycolysis. Among the three main pathways, alcoholic fermentation is considered the most important, since about 92% of the pyruvate generated through glycolysis is directed to ethanol production, and only 7% to lactate and 1% to alanine pathways (Kato-Noguchi, 2006). Alcoholic fermentation is strongly activated in rice during germination under oxygen deprivation, as reflected in the induction of the key enzymes, pyruvate decarboxylase (PDC), alcohol dehydrogenase (ADH) and aldehyde dehydrogenase (ALDH). The rest of this review will discuss some of the enzymes involved in carbohydrate metabolism that are differentially activated in contrasting rice genotypes germinating under anaerobic conditions, especially those involved in the two critical steps where oxygen is limiting. Alanine metabolism under anoxia is also relevant in rice, especially as related to glutamine and glutamate synthesis for aminoacid accumulation (Reggiani et al., 2000); however, this will not be discussed in this review.

### Role of anaerobic respiration in seeds germinating under aerobic and anaerobic conditions

In aerobic (normal) conditions, seeds of cereal crops like wheat and rice, have some hypoxic or even anoxic tissues where respiration and therefore energy production become limiting during germination (van Dongen et al., 2004). Rice seeds germinating in aerobic conditions obtain energy from lipid and carbohydrate catabolism. Lipid mobilization is an important part of seed germination in aerobic conditions. Lipid degradation, β-oxidation, glyoxylate cycle and related enzymes were found in germinating rice seeds after 24 h of imbibitions (He et al., 2011). During carbohydrate catabolism, both respiration and alcoholic fermentation are induced, as observed by transcript analysis of particular enzymes in germinating rice seeds (Howell et al., 2006, 2009). This is supposedly to regulate respiration and avoid internal anoxia, which would jeopardize embryo growth (Zabalza et al., 2009). Alcohol dehydrogenase (*adh*) deficient *rad* mutant accumulated pyruvate, which could accelerate respiration and lead to anoxia and irreparable cell damage. The anaerobic pathway may therefore be needed under aerobic conditions to avoid this internal crisis. Also, during the first hours of germination between 0 and 48 h after imbibition, mitochondria will be developed and respiration begins. Until 48 h after imbibition, the pyruvate dehydrogenase (PDH) complex and the tricarboxylic acid (TCA) cycle enzymes are not fully active, and energy is obtained through an alternative NADH dehydrogenase (Howell et al., 2006). A similar pattern was found for the cytochrome c, suggesting that aerobic respiration does not start until 48 h after seed imbibition. However, under submergence, limited oxygen poses several bottlenecks in the aerobic carbohydrate metabolism. The two major bottlenecks are degradation of starch and complex carbohydrates into simple sugars and pyruvate catabolism to generate energy.

#### First bottleneck: degradation of starch and complex carbohydrates to soluble sugars

Continuation of carbohydrate metabolism is essential for seed germination and seedling establishment under submergence as has been highlighted in several studies (e.g., Ella and Setter, 1999; Ismail et al., 2009; Magneschi and Perata, 2009). Seeds with large carbohydrate storage reserves have an adaptive advantage under stress and pre-germinated seeds have an advantage over non-germinated seeds when oxygen is limiting (Ella et al., 2011), suggesting that the early processes during germination, especially cell division, are probably more sensitive to low oxygen stress. The only cereal with the necessary set of enzymes needed to break down starch under submergence is rice (Atwell and Greenway, 1987; Perata et al., 1992, 1993; Ismail et al., 2012). Furthermore, Ismail et al. (2009) observed that tolerant rice genotypes had greater ability to break down starch into soluble sugars than sensitive genotypes and therefore, their coleoptiles could sustain higher elongation rates. They further showed that tolerant “Khaiyan” also sustained higher soluble sugar concentrations in germinating seeds. It was later demonstrated that these tolerant landraces also store relatively more soluble sugars in their endosperm than sensitive genotypes (Ismail et al., 2012).

This initial step in starch catabolism provides soluble sugars for glycolysis and subsequent metabolic processes. Seemingly, this step is the one that mostly hinders the ability of intolerant rice varieties and other cereals such as wheat, to germinate under anaerobic conditions (Ismail et al., 2009; Magneschi and Perata, 2009). Two enzymes are considered important for carbohydrate catabolism in rice seeds germinating under low oxygen: sucrose synthase for the breakdown of sucrose, and α-amylases for starch breakdown. Sucrose synthase is functional in rice under submerged conditions at the same rate as in air, but not in wheat and barley seedlings (Magneschi and Perata, 2009). However, there is no apparent difference in the activity of sucrose synthase in seeds of tolerant and sensitive rice genotypes germinating under submergence (Ismail et al., 2009). Therefore, variation in the activity of this enzyme might not have an adaptive significance. Maintenance of higher activity of α-amylases, on the other hand, has been widely reported as an important step in carbohydrate catabolism under submergence, when oxygen is suboptimal (Guglielminetti et al., 1995; Perata et al., 1997; Hwang et al., 1999), and higher activity was observed in seeds of tolerant genotypes germinating in flooded soils. *RAmy3D* was differentially expressed in the tolerant genotype “Khaiyan” compared with the sensitive IR42. In addition, *RAmy3D* expression correlated positively with coleoptile elongation and seedling survival, especially in tolerant genotypes (Ismail et al., 2009, 2012). Since GA is apparently not produced in germinating seeds under anoxia, the GA activated α-amylase *RAmy1A* was not functional. Instead, *RAmy3D* was active in tolerant genotypes under anoxia and this is probably because its activation is not regulated by GA as for its aerobic counterpart *RAmy1A* (Loreti et al., 2003a). That the expression of *RAmy3D* is GA independent was established in a GA mutant capable of germination under anoxia (Loreti et al., 2003b). Additional studies involving expression analysis using rice coleoptiles growing under anoxia also showed positive correlation between the expression of *RAmy3D* and starch degradation (Lasanthi-Kudahettige et al., 2007). This gene is activated during the first 2 days after submergence (Loreti et al., 2003a; Lasanthi-Kudahettige et al., 2007). Collectively, these studies suggest that this gene is important for tolerance of anaerobic conditions during germination in rice. However, since no *RAmy3D* mutant studies have been published so far, we can only speculate how indispensable the enzyme is for tolerance of submergence during germination.

*RAmy3D* is a glucose and sucrose sensor that is apparently activated by the protein kinase *SnRK1A* under glucose starvation (Lu et al., 2007). The *SnRK1A* in turn, gets activated by calcineurin B-like protein kinase *CIPK15* (Lee et al., 2009; Kudahettige et al., 2010). Its target, the Calcineurin B-like protein 5 (CBL5) is upregulated both at the protein and mRNA levels under anoxia. CBLs contain the common calcium binding domain motif EF-hand. The up-regulation of CBL5 suggests that it may interact with CIPK15 to activate downstream signaling process involved in germination under anoxic conditions (Sadiq et al., 2011). Park et al. (2010) showed that regulation of α-amylases was disrupted by the inhibition of oxidative phosphorylation. They suggested that the removal of repression of the sugar effect on the transcription of *CIPK1* is required for the activation of *SnRK1A* and in turn, the activation of *RAmy3D* during anaerobic germination of rice. Interestingly, *CIPK15* has been shown to act beyond germination. It can sustain its activity until maturity under partial submergence (Lee et al., 2009). However, its role during complete submergence at the vegetative stage is not yet clear, and could either complement or conflict with the *SUB1A* pathway (Kudahettige et al., 2010).

#### Second bottleneck: metabolism of pyruvate

Three main pathways were identified for anaerobic catabolism of carbohydrates: alcoholic fermentation, lactate fermentation and alanine fermentation. Alcohol fermentation is by far considered the main alternative pathway under anaerobic conditions (Ricard et al., 1994) as the metabolic reactions occurring via the alcoholic or ethanolic fermentation does not decrease the cell pH (Geigenberger et al., 2003). The alcoholic fermentation pathway will be discussed in more details for the remainder of this review.

The first reaction in the alcoholic fermentation pathway is the conversion of pyruvate generated during glycolysis to acetaldehyde by the enzyme pyruvate decarboxylase (PDC, EC 4.1.1.1.). During this irreversible reaction, a CO_2_ molecule is generated. Acetaldehyde is metabolized in one of two ways; conversion to ethanol by alcohol dehydrogenase (ADH, EC 1.1.1.1.) or conversion to acetate by mitochondrial aldehyde dehydrogenase (mALDH, EC 1.2.1.3.). When external oxygen decreases (hypoxia or anoxia), aerobic respiration is inhibited in a coordinated response that decreases the adenylate status, the TCA cycle and glycolysis (Geigenberger et al., 2003). The response to low oxygen (a drop below 5%) has been found to act in two phases, firstly a pre-adaptation response to maintain energy levels through *pdc1*, *pdc2*, *adh1*, and *aldh1* genes, and secondly detoxification of reactive oxygen species through e.g., catalase, superoxide dismutase, ascorbate peroxidase, monodehydroascorbate reductase, glutathione reductase, and superoxide dismutase (Klok et al., 2002). Expression of ADH is sensitive to oxygen concentrations (van Dongen et al., 2009), and is usually the most up-regulated enzyme of the fermentative pathway. ADH quickly metabolizes acetaldehyde to reduce the risk of acetaldehyde-mediated cytotoxicity, and to generate NAD^+^ for glycolysis. Ismail et al. (2009) observed that the activity of ADH reached 100 times that of ALDH and about 10 times that of PDC in the tolerant genotype “Khao Hlan On” compared with the sensitive “IR42” (Table 2; Ismail et al., 2009; A.M. Ismail, unpublished).

**Table 2:** **Activities (units min^-1^ mg^-1^ protein) of alcohol dehydrogenase (ADH) and pyruvate decarboxylase (PDC) enzymes in the tolerant “Khao Hlan On” and the sensitive “IR42” from day 0 (dry seeds) to day 10 under aerobic conditions and under 10 cm of submergence (hypoxia) in darkness**.

	**ADH activity**				**PDC activity**			
**Day**	**Khao Hlan On**		**IR42**		**Khao Hlan On**		**IR42**	
	**Hypoxia**	**Aerobic**	**Hypoxia**	**Aerobic**	**Hypoxia**	**Aerobic**	**Hypoxia**	**Aerobic**
0	5	5.0	4.0	4.0	0.5	0.50	0.40	0.40
1	14	6.0	5.0	5.0	1.3	0.70	0.70	0.70
2	18	5.0	6.0	5.0	2.0	0.60	1.00	0.60
3	20	5.0	7.0	4.0	2.2	0.50	0.90	0.50
4	21	4.0	8.0	4.0	2.5	0.55	1.00	0.50
5	23	3.0	8.5	3.0	3.0	0.40	0.90	0.40
6	25	3.0	10.0	3.0	2.7	0.40	0.80	0.40
7	24	2.5	9.0	2.5	2.2	0.30	0.85	0.30
8	23	2.0	9.0	2.0	2.2	0.30	0.80	0.30
9	24	1.0	9.5	2.0	2.3	0.35	1.00	0.35
10	24	1.0	10.0	1.5	2.2	0.40	1.00	0.30

*Data adapted from Ismail et al. (2009)*.

Most reactions of the ethanolic fermentation seem to be substrate regulated to avoid buildup of toxic products, and also to regulate energy production and consumption (Gibbs and Greenway, 2003). For example, depletion of acetaldehyde through the two pathways of ADH and ALDH can swing the ADH reaction in the opposite direction to generate acetaldehyde, the replenishment of which can regulate the activity of PDC. Thus, ADH can indirectly regulate the pyruvate dehydrogenase (PDH) bypass under submergence by substrate regulation of acetaldehyde. Mitochondrial ALDH is activated instead of the cytoplasmic ALDH to avoid competition for NAD^+^ with glycolytic enzymes, as the cell stress regulation mechanisms involving energy saving are activated under submerged conditions (Nakazono et al., 2000). The mitochondria sequestered ALDH is not substrate inhibited.

A desaturation mechanism involving pyruvate consumption was invoked to justify the up-regulation of PDC and ADH (Ismail et al., 2009). Similarly the ADH activity was induced in germinating seeds of other cereals, but the highest induction was reported in rice (Guglielminetti et al., 2001; Shingaki-Wells et al., 2011). In anoxia-sensitive maize and pea the ADH activity increase was mostly in roots, whereas in tolerant species like rice and *Echinochloa* it increased in coleoptiles (Cobb and Kennedy, 1987). However, this trend was not found in barley (Kato-Noguchi, 1999).

Plants mutated for or overexpressing ADH and/or PDC can be used to more clearly establish the role of alcohol fermentation during hypoxia or anoxia. For example, the rice cultivar “Taipei 309” over-expressing PDC had higher tolerance of anoxia due to an increase in alcohol metabolism (Quimio et al., 2000). However, over-expression of both PDC and ADH in the same cultivar did not improve anoxia tolerance, probably because of acetaldehyde accumulation (Rahman et al., 2001). Other studies with mutations in *adh* (Matsumura et al., 1995, 1998; Conley et al., 1999; Takahashi et al., 2011; Tougou et al., 2012) suggest that lack of ADH1 creates an energy deficit that affects seed germination, inducing responses similar to that of germination under submergence. Takahashi et al. (2011) concluded that the shortage of energy in the *rad* mutant when germinated under complete submergence affected both cell division and cell extension.

***The formation of acetyl CoA and the PDH bypass***. The most favorable pathway in anaerobic metabolism is probably the production of acetate to remove toxic acetaldehyde and to recycle carbon for use in other pathways such the glyoxylate cycle, and to feed TCA cycle intermediates. Acetate could then accumulate or be converted into TCA cycle intermediates like malate or citrate (Yamashita and Fujiwara, 1966). Under aerobic conditions, pyruvate is converted to acetyl-CoA by the pyruvate dehydrogenase enzymatic complex. However, the first acting enzyme of the complex is not functional under anaerobic conditions, preventing direct production of acetyl-CoA from pyruvate. Thus, an alternative pathway has been suggested to involve the conversion of pyruvate to acetyl-CoA by PDC, mALDH and acetyl-CoA synthase (ACS, EC 6.2.1.1). When oxygen levels decrease NADH accumulates, the pyruvate dehydrogenase complex unit is subsequently inhibited (Yamamoto, 1966) and pyruvate does not feed the TCA cycle, since the complex is feedback regulated by NADH (Randall and Miernyk, 1990). The unit of the complex being inhibited is E_1_—pyruvate dehydrogenase (PDH) (Howell et al., 2009). This resulted in reduction of energy production starting after about 2 h of submergence (Vigeolas et al., 2003).

**Figure 2:**
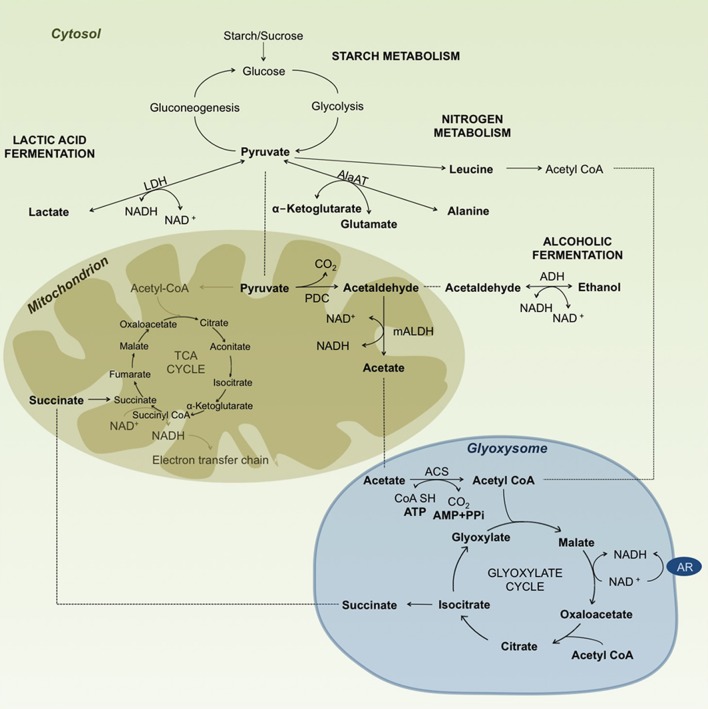
**Diagram summarizing carbohydrate metabolism under anoxic/hypoxic conditions in rice**. Enzyme abbreviations: AlaAT, alanine aminotransferase (EC 2.6.1.2); LDH, lactate dehydrogenase (EC 1.1.1.27); PDC, pyruvate decarboxylase (EC 4.1.1.1); ADH, alcohol dehydrogenase (EC 1.1.1.1); mALDH, mitochondrial aldehyde dehydrogenase (EC 1.2.1.3); ACS, acetyl-CoA synthetase (EC 6.2.1.1); AR, ascorbate-free radical reductase (EC 1.6.5.4). ······indicates substrate movement and—indicates substrate conversion/modification. Reaction substrates are highlighted in bold letters.

The conversion of acetaldehyde to acetyl-CoA by ALDH and ACS results in the consumption of one NAD^+^, one CoA and one ATP molecule, generating a CO_2_ molecule, NADH, AMP and PPi. This 3 step alternative pathway results in a loss of two carbon molecules in the anaerobic reaction compared with the 1 step aerobic reaction of the pyruvate dehydrogenase complex. In *Echinochloa* coleoptiles, it has been shown that lipid synthesis and accumulation continue under anoxic conditions (Kennedy et al., 1991). That could be explained by the continuation of fatty acid synthesis through the PDC-ALDH-ACS pathway (Figure 2). These authors also found that the mitochondria maintain their integrity and functionality under anoxia. This hypothesis of the PDC-ALDH-ACS pathway has been suggested to be operational in plants as in tobacco (Tadege et al., 1997, 1999), rice (Lu et al., 2005) and *Arabidopsis* (Wei et al., 2009). The pathway was well-studied in yeast, *Saccharomyces cerevisiae* (Klein and Jahnke, 1979; Pronk et al., 1994; van den Berg and de Steensma, 1995). Lu et al. (2005) submerged 10-day-old seedlings of an *indica* variety “Guangluai 4” for durations of 12 h to 3 days, and found an increase in ACS activity in submerged seedlings, confirming the conversion of acetate into acetyl-CoA under submergence. Moreover, they reported an increased expression of *ALDH2a* during the first hours of submergence and that of *ALDH2b* after 2 days of submergence.

Other alternatives for acetyl CoA production may arise from the nitrogen metabolism of ketogenic aminoacids like leucine (Leu), isoleucine (Ile), tyrosine (Tyr), phenylalanine (Phe) and tryptophane (Trp) (Figure 2). Accumulation of Leu has been reported to increase under anaerobic conditions in rice (Fan et al., 1997; Narsai et al., 2009; Shingaki-Wells et al., 2011), providing the needed substrate for acetyl CoA production under starved conditions by bypassing the steps of the TCA cycle. Reports on other aminoacids like Tyr, Phe and Trp were inconsistent, and showed either an increase or decrease in different studies. For example, Narsai et al. (2009) reported an increase in Phe and Tyr, whereas Shingaki-Wells et al. (2011) found a decrease in Tyr and no changes in Phe, but also found an increase in Trp, which was not observed in the Narsai et al. (2009) study.

***The importance of ALDH in anaerobic germination***. Several studies have linked increase in ALDH activity to tolerance of submergence in rice (Nakazono et al., 2000; Tsuji et al., 2003). These studies reported that the mitochondrial ALDH family in particular had the most significant increase in activity in varieties able to germinate under anaerobic conditions (Nakazono et al., 2000; Tsuji et al., 2003). These studies also concluded that *ALDH2a* is more responsive to anaerobiosis and could therefore play a more important role in detoxifying acetaldehyde (Meguro et al., 2006). Similar observations were recently made in a proteomics study by Sadiq et al. (2011) using seeds of the variety “Arborio” imbibed prior to submergence. Lasanthi-Kudahettige et al. (2007) found that *ALDH2a* (*Os02g49720*) was up-regulated by 11-fold and *ALDH2b* (*Os06g15990*) was down-regulated by 22 fold in Nipponbare seeds subjected to anoxia since imbibition. A study by Lu et al. (2005) using the *Oryza sativa* indica cultivar “Guangluai 4” found that the expression of *ALDH2a* was activated between 12 and 72 h after the initiation of anoxia, whereas *ALDH2b* was expressed 48 h after the start of the treatment. Still in another study, Wei et al. (2009) tested *Arabidopsis* single, double and triple mutants for the three family 2 ALDH genes with ^14^C-ethanol. They developed *Arabidopsis* mutants each lacking one gene (cytosolic *aldh2C4* alleles 1 and 2; mitochondrial *aldh2B4* alleles 1 and 2 and *aldh2B7*) and their possible combinations, and subjected the mutants and wild types to different stress treatments, including 6 h of hypoxia. They found no differences between the mutants for *aldh2C4* and the wild type, suggesting that the cytosolic enzymes are not relevant for the PDC-ALDH-ACS pathway. However, they found lower ^14^C-ethanol incorporation in the mutants lacking ALDH activity, thus suggesting that the PDH bypass cycle functions in seedlings of *Arabidopsis* when the PDH enzyme is not functional. Among the three ALDH genes, they found that the main ALDH involved in the PDH bypass is *ALDH2B4*, since the ^14^C-ethanol incorporation rate was the lowest in the *aldh2B4* mutant. They did not, however, find any significant phenotype with the different ALDH mutants (Wei et al., 2009).

Differences in gene expression between the varieties used in these studies may be due to various reasons as shown in Dubois et al. (2011). For example, the level of tolerance may be variable between varieties, even though they have a common phenotype (coleoptile elongation under submergence). Some varieties are either moderately tolerant or sensitive of anoxia during germination; however, they have the ability to extend their coleoptiles in hypoxic conditions (A. M. Ismail, personal communication). Moreover, some varieties showed moderate elongation of their coleoptiles under hypoxia or anoxia imposed prior to seed imbibition and these genotypes may use different survival strategies than that observed in “Khao Hlan On,” “Khaiyan” and other tolerant genotypes. Also sensitive varieties may have different responses at the molecular level depending on their strategy, i.e., escape or quiescence. For example in a study with the two tolerant rice genotypes, “Khao Hlan On” and “Khaiyan,” and two sensitive genotypes, “FR13A” and “IR42,” dry seeds were germinated in 10 cm of water in darkness. Increased activity of both PDC and ADH was observed in all genotypes under submerged (hypoxic) conditions. However, PDC and ADH activities were substantially higher in the tolerant genotypes than in the sensitive ones under submerged conditions (Ismail et al., 2009; Table 2). A similar trend was noticed for ALDH2 (A.M. Ismail, pers. communication). This higher activity in the tolerant genotypes under submerged conditions would probably indicate that tolerance of hypoxic conditions during germination is related to the capacity of the plant to metabolize acetaldehyde and recycle carbon. It has previously been reported that alcoholic fermentation toxicity is not due to the ethanol accumulation per se, but rather to the accumulation of acetaldehyde (Perata and Alpi, 1993, in carrot).

The role of ALDH in detoxifying reactive oxygen species (ROS) has also been suggested in animal systems (Ohsawa et al., 2003; Chen et al., 2007; Xue et al., 2012). These studies found a relationship between ROS accumulation and reduced activity of ALDH2, and vice versa, reduced ROS with increasing ALDH2 activity. They also found that ALDH2 is ethanol activated, leading to the activation of a particular NO synthase in humans. This action happens by reducing ROS accumulation (Xue et al., 2012). Moreover, Chen et al. (2007) suggested that oxidative stress inhibits ALDH2 either by oxidizing the enzyme or its cofactors. In plants, there are some enzymes known to use fermentation products to produce ROS—xanthine oxidase can use acetaldehyde as an electron donor and generate O^−^_2_ and H_2_O_2_ (Harrison, 2002). Therefore, the direct action of ALDH may not detoxify ROS, but reduction in its production by metabolizing acetaldehyde may regulate ROS content. Additionally, ALDH could inhibit enzymes related to lipid peroxidation and scavenging of ROS (Kotchoni et al., 2006). Since ALDH activity is much higher in “Khao Hlan On” under submergence while ROS concentrations are lower in the same genotype, we hypothesize that ALDH has a detoxifying function for both acetaldehyde and ROS. More research is under way to provide further evidences for the role of ALDH in these processes.

Experiments at the protein level using cultivar “Arborio” (Sadiq et al., 2011) showed the expression of both ALDH2a and ALDH2b under submerged conditions. Different trends were observed for “Khao Hlan On” and “IR42.” “Khao Hlan On” maintained the same levels of both ALDH2a and ALDH2b under submergence, while “IR42” had almost negligible amounts of either protein after day 5 (A.M. Ismail, personal communication). Both rice ALDH2a and ALDH2b and *Arabidopsis* ALDH2B4 and ALDH2B7 were classified very closely in group 2 in a phylogenetic analysis by Kotchoni et al. (2010). This classification showed that rice ALDH2a (OsALDH2B5) and ALDH2b (OsALDH2B1) had similar evolution pathway as that of *Arabidopsis* ALDH2B4 and ALDH2B7. Wei et al. (2009) found that ALDH2B4 is more relevant to the PDH bypass than ALDH2B7. Apparently, further studies are needed to affirm the role of these enzymes in tolerant rice genotypes.

***Regulation of alcohol fermentation and its enzymes***. The up-regulation of alcoholic fermentation under submergence could be associated with a decrease in oxygen (Nakazono et al., 2000; Ismail et al., 2012) or in the energy levels (Zabalza et al., 2009). It has also been suggested that *Adh1* and *Pdc1* genes are regulated by the concentrations of cytosolic calcium induced by low oxygen conditions (in maize, Subbaiah et al., 1994; in rice, Nakazono et al., 2000; Tsuji et al., 2000). However, the latter authors found that ALDH2a does not share the same mechanism, and there might be two signaling mechanisms under submergence: oxygen dependent and an oxygen independent one. Another hypothesis suggested that the activation of alcoholic fermentation is due to higher pyruvate concentration (discussed in Bailey-Serres and Voesenek, 2008). Based on Roberts et al. (1984) the activation of these enzymes in maize is caused by a decrease in cytoplasmic pH. The regulation of PDC and ADH activities by cellular pH under anoxia was also hypothesized earlier for rice (Davies, 1980), but it was later suggested that alcoholic metabolism is most likely regulated by pyruvate concentration than by cytosolic pH (Kato-Noguchi, 2006). Zabalza et al. (2009) also suggested that high pyruvate concentration activates alcoholic fermentation. They also argued that ADH expression is probably regulated by low oxygen, but later modified by the energy status of the tissue where it is expressed.

Some enzymes have isozyme forms in different organelles and compartmentalization of these isozymes facilitates continuation of some necessary processes in a sequestered manner. For example, the cytosolic enzyme ALDH1 is inhibited under submergence, whereas the mitochondrial enzyme ALDH2 is activated. Carystinos et al. (1995) suggested that the enzyme under stress is less effective than the enzyme active in the absence of stress, because of less efficient translation, less enzyme stability or lower activity. Results similar to those observed in rice showing higher up-regulation of the enzymes involved in alcoholic fermentation in the tolerant genotype were also reported in tolerant *Echinochloa formosensis*, where both PDC and ALDH were activated at much higher rates than in the sensitive *E. praticola* under submergence (Fukao et al., 2003). We recently observed that the 2 isozymes of ALDH are apparently co-regulated by the same activation/deactivation factor at least at the protein level (A.M. Ismail, personal communication), which seems to contradict results from Tsuji et al. (2003) and Sadiq et al. (2011), who suggested that different mechanisms are probably involved in the regulation of ALDH2a vs. ALDH2b, since each enzyme is translated at a different time point. Higher concentration of ALDH2 under submergence suggests this enzyme is probably involved in the tolerance of rice genotypes of submergence during germination and early seedling growth, by catalyzing acetaldehyde detoxification, but further studies will be carried out to affirm this conclusion.

Another important regulatory mechanism relates to substrate concentration and saturation of the different enzymatic reactions involved (Tadege et al., 1999). The higher activities of ADH and PDC observed in the tolerant rice genotypes “Khaiyan” and “Khao Hlan On” than in the sensitive genotypes (Ismail et al., 2009), could possibly be explained by the ability of these tolerant genotypes to metabolize acetaldehyde to acetate, and thus extend the saturation point for acetaldehyde accumulation, which would otherwise be cytotoxic. Besides, tolerant genotypes might have evolved efficient mechanisms to either remobilize or sequester acetaldehyde to avoid both saturation and toxic build-up in the cell cytoplasm. It is also noteworthy mentioning a possible role of ALDH in reducing the ethanol accumulation in cells to avoid post-anoxic damage upon re-aeration. This could involve reactions of ethanol mediated by catalases forming hydrogen peroxide and also oxidation to acetate. As depicted in Meguro et al. (2006), ethanol accumulated in cells during submergence is oxidized to acetaldehyde or hydrogen peroxide when oxygen becomes available upon shifting to aerobic conditions.

#### Other bottlenecks: the tricarboxylic acid cycle and beyond

Some enzymatic changes also occur in the tricarboxylic acid (TCA) cycle leading to important changes favoring plant survival of anaerobic conditions (Fox and Kennedy, 1991; Fox et al., 1994; Fan et al., 2003; Howell et al., 2006; Nakamura et al., 2012). Fox and Kennedy (1991) reported that 2-oxoglutarate dehydrogenase activity decreased under anoxia and the conversion of 2-oxoglutarate to succinate was identified as the limiting step in the TCA cycle under these conditions. They also found that enzyme activities generally declined after 7 days of anoxia. Conversely, a recent study involving metabolic profiling of rice and wheat coleoptiles under anoxia reported higher concentrations of succinate and also citrate, aconitate, and fumarate under anaerobiosis (Shingaki-Wells et al., 2011). These authors further observed synthesis of the amino acids lysine, methionine, threonine and isoleucine, indicating that the TCA cycle is probably functioning to a certain extent. On the other hand, Wei et al. (2009) suggested that acetyl-CoA is likely converted into fatty acids in the plastid in *Arabidopsis*.

Based on Lu et al. (2005), acetyl-CoA enters the glyoxylate cycle in rice coleoptiles under anaerobic conditions. This cycle is a short version of the TCA cycle that takes place in the glyoxysome, where isocitrate is converted to glyoxylate by isocitrate lyase and glyoxylate to malate by malate synthase. By the action of these two enzymes, the cycle omits two steps of the TCA cycle: from isocitrate to α-ketoglutarate, and then to succinyl-CoA, which is then converted to succinate. By skipping these two steps, the glyoxylate cycle avoids the loss of two carbon molecules, thus giving a net of 4-carbon malate or oxaloacetate (Figure 2). To prove these processes, the authors analyzed mRNA transcripts of the different enzymes involved, and they reported higher expression of isocitrate lyase and malate synthase under submergence. They then concluded that this is the pathway that rice seedlings use to keep their metabolism ongoing under submerged conditions (Lu et al., 2005). Fan et al. (2003) found evidence that the TCA cycle is maintained at least up to the α-oxoglutarate step in rice. However, analyzing the rate of malate production, they also found that malate was mainly being produced by the glyoxylate cycle. Malate could be converted to succinate in this cycle, which could then enter the mitochondrion. Fan et al. (2003) tested pre-germinated seeds of the japonica rice variety “M201” after submergence in darkness. They then followed the metabolic reactions taking place under hypoxia *in vivo* with ^13^C nuclear magnetic resonance. In this scenario, both cycles maintain carbohydrate catabolism and also support protein biosynthesis. A similar hypothesis was proposed by Tadege et al. (1999) in tobacco pollen. They suggested that both the TCA and glyoxylate cycles were functional, and that the two cycles were simultaneously generating substrates and energy for the cells. In the tolerant “Khao Hlan On,” effective ethanol fermentation involving pyruvate decarboxylase, alcohol dehydrogenase and, more importantly, aldehyde dehydrogenase would confer an adaptive advantage for germination under submerged conditions. That could possibly be coupled with the reducing power of the glyoxylate cycle, which could sustain both carbohydrate catabolism and protein biosynthesis.

### The role of fatty acid β-oxidation in aerobic conditions

Glyoxylate cycle in the glyoxysomes carries out lipid metabolism during germination (Donaldson et al., 2001). Conversion of triacylglycerols into sugars by β-oxidation, in the glyoxysomes for energy supply during germination is particularly well-studied in oilseed plants. Rice seeds contain an average of 20% lipids in bran and germ, nearly half that of an oilseed, and could be a possible source of energy under submerged conditions. This pathway can also produce more acetyl-CoA besides generating net energy. This would generate substrates via the glyoxylate cycle to continue with the partial TCA cycle and feed gluconeogenesis metabolism. In turn, it would replenish carbon in the process, thus making metabolism more efficient. The degradation of triglycerides into free fatty acids and glycerol is supposedly carried out under anoxia by ACS (Lu et al., 2005). Moreover, 28 transcripts of proteins involved in lipid metabolism have been found in comparative studies between rice seeds germinating under aerobic and anaerobic conditions (Narsai et al., 2009). However, lipid metabolism in rice germinating under anoxia or hypoxia seems to be shifted toward biosynthesis, since a large proportion of the ATP generated is allocated to maintain membrane integrity (Edwards et al., 2012). In addition, fatty acids do not seem to serve as electron acceptors under submergence in rice coleoptiles (Generosova and Vartapetian, 2005). Other glyoxysome ezymes up-regulated under anoxia, such as the ascorbate peroxidase (metabolizes H_2_O_2_ into H_2_O) and monodehydroascorbate reductase (regenerates NAD^+^ from NADH; Donaldson et al., 2001) could have a role in the detoxification of reactive oxygen species (Lasanthi-Kudahettige et al., 2007). Higher activities of superoxide dismutase and catalase were also observed in seeds of tolerant rice genotypes germinating in water (Ella et al., 2011). This was associated with lower lipid peroxidation and higher survival. The definitive association of variation in expression or activities of these genes with flood tolerance in rice awaits further investigation.

## Conclusions and perspectives

Rice is the only cereal capable of germination under submerged conditions, with substantial variation in tolerance of submergence at this stage, within cultivated varieties and landraces. Rice has been used extensively in studies of tolerance of anoxia and hypoxia and also used as a model plant when comparing different crop species. However, the regulatory and signaling mechanisms controlling tolerance of anaerobiosis during germination in tolerant rice genotypes are yet to be revealed. Some progress has been made in uncovering the major traits associated with tolerance of submergence during germination in some rice genotypes, some of which provided evidence supporting the role of the PDC-ALDH-ACS pathway in tolerance. Further studies are needed to elucidate the critical roles of the enzymes of carbohydrate, fatty acid and energy generating metabolic processes associated with tolerance. For example, RAmy3D and ALDH2 are good candidates for studies involving gene silencing or knock-out and complementation. Furthermore, identifying functional alleles associated with tolerance in major anaerobic pathways will help in speeding up breeding programs to develop tolerant rice varieties and varieties of other crops for which waterlogging is a serious problem during crop establishment as in wheat, barley and maize. These findings could also facilitate developing strategies that can help in managing and controlling aquatic weeds and weeds that are becoming problematic in paddy fields because of the evolution of new ecotypes that are more adapted to flooded conditions (Ismail et al., 2012).

Despite the efforts devoted for understanding the different mechanisms associated with tolerance of submergence during germination, development of improved varieties has not yet been possible. Steady progress has, however, been made while bottlenecks continue to be unveiled. One difficulty is the lack of homogeneity of data in the studies available. For example, there is currently little information on integrative data from studies using different approaches or addressing different mechanisms for germination under submerged conditions. Many studies on germination under submergence compare rice to other crops, with rice being the tolerant species. In some cases, studies used rice genotypes that are not particularly tolerant of anaerobic conditions during germination. Only a limited number of comparative studies have included reasonably tolerant rice genotypes, meaning those that germinate under water and develop into mature plants with little or no negative effects on crop establishment. Moreover, the experimental plans for such studies lack a unified approach in most cases, where different testing protocols are followed. Importantly, these protocols do not reflect field conditions, thereby negating the value of the results obtained toward varietal development. These are important obstructs since they lead to a major divergence of opinions in the classification of genotypes for tolerance of submergence during germination. Furthermore, germination under submergence is a complex trait based on current evidences, which suggests that differences exist between anoxic and hypoxic treatments and different rice genotypes probably respond differently to such treatments. Environmental factors also seem to interact with tolerance, e.g., floodwater temperature, seed and seedbed conditions (Ella et al., 2010, 2011). Hence, variations in the experimental conditions may lead to different results, which are not necessarily contradictory but basically not applicable in standardizing genotype-specific tolerance mechanisms. Future research should be based on appropriately selected genotypes and experimental conditions to provide more insightful results on the mechanisms of tolerance of submergence during germination.

### Conflict of interest statement

The authors declare that the research was conducted in the absence of any commercial or financial relationships that could be construed as a potential conflict of interest.
